# Prevalence and diagnostic stability of ADHD and ODD in Turkish children: a 4-year longitudinal study

**DOI:** 10.1186/1753-2000-7-30

**Published:** 2013-08-07

**Authors:** Eyüp Sabri Ercan, Rasiha Kandulu, Erman Uslu, Ulku Akyol Ardic, Kemal Utku Yazici, Burge Kabukcu Basay, Cahide Aydın, Luis Augusto Rohde

**Affiliations:** 1 Department of Child and Adolescent Psychiatry, Faculty of Medicine, Ege University, Izmir 35100, Bornova, Turkey; 2 Child Psychiatric Division, Hospital de Clinicas de Porto Alegre, Federal University of Rio Grande do Sul, Brazil and the National Institute for Developmental Psychiatry, São Paulo, Brazil

**Keywords:** ADHD, ODD, Epidemiology, Prevalence

## Abstract

**Background:**

This study was designed to assess the prevalence of Attention-Deficit/Hyperactivity Disorder (ADHD) and Oppositional Defiant Disorder (ODD) in a representative sample of second grade students from a country in a region where no previous rates are available (Turkey). The second aim is to evaluate the differences in ADHD and ODD prevalence rates among four different waves with one-year gap in reassessments.

**Method:**

Sixteen schools were randomly selected and stratified according to socioeconomic classes. The DSM-IV Disruptive Behavior Disorders Rating Scale (T-DSM-IV-S) was delivered to parents and teachers for screening in around 1500 children. Screen positive cases and matched controls were extensively assessed using the K-SADS-PL and a scale to assess impairment criterion. The sample was reassessed in the second, third and fourth waves with the same methodology.

**Results:**

The prevalence rates of ADHD in the four waves were respectively 13.38%, 12.53%, 12.22% and 12.91%. The ODD prevalence was found to be 3.77% in the first wave, 0.96% in the second, 5.41% in the third and 5.35% in the fourth wave. Mean ODD prevalence was found to be 3.87%.

**Conclusions:**

The prevalence rates of ADHD in the four waves were remarkably higher than the worldwide pooled childhood prevalence. ADHD diagnosis was quite stable in reassessments after one, two and three years. A mean ODD prevalence consistent with the worldwide-pooled prevalence was found; but diagnostic stability was much lower compared to ADHD.

## Background

Attention-Deficit/Hyperactivity Disorder (ADHD) and Oppositional Defiant Disorder are among the most common psychiatric disorders of childhood either in community or clinical samples [1-3]. Moreover, they co-occur much more frequently than expected by chance [4]. Both disorders are related with substantial impairment and can be precursors of CD, severe delinquent behavior and substance use disorders [2,5].

As in any medical conditions, the development of health strategies directed to early diagnosis and treatment of ADHD depends on robust epidemiological data [6]. It is important to note that a huge variability in prevalence rates are detected among studies [6]. Although previous literature clearly suggests that heterogeneity in prevalence rates of the disorder is associated with methodological differences among studies [7], there are yet concerns that ADHD prevalence might be exaggerated in some environments [8]. Faraone et al. [9] stated that, the predominance of American research into this disorder over the past 40 years has led to the impression that ADHD is largely an American disorder and is much less prevalent elsewhere. This impression was reinforced by the perception that ADHD may stem from social and cultural factors that are most common in American society. Moreover, some regions of the world were underrepresented in the most comprehensive review of the ADHD epidemiology in youths [7]. The only significant differences in prevalence rates were found between North America and both Middle East and Africa. However, as stated by the authors, these differences might be an artifact of the small number of studies in those regions (4 from Middle East and 4 from Africa) introducing instability in analyses. As stated by Polanczyk and Jensen [6], more studies conducted in areas with scarcity of data with better methodologies are needed to really expand the knowledge on the worldwide prevalence of the disorder.

Despite its clinical relevance, surprisingly very little data is available about ODD prevalence. One possible reason for this is the fact that several studies implemented the evaluation of ODD in combination with CD under a category named “conduct problems”. This situation has its roots in a usual tendency to view ODD under the umbrella of CD. With rare exceptions, the epidemiology, comorbidity pattern and course of ODD were investigated in combination with CD [10]. However recent studies showed that ODD could be separated both from CD and normal child behavior and must be studied independently to be well understood [11].

Studies on ODD prevalence produced a wide range of rates from 2 to 15% [12-14]. Nock et al. [11] and Maughan et al. [15] suggested that the important variation in ODD prevalence might be related to the use of non-representative samples and inconsistent diagnostic approaches in the studies. Very recently, the characteristics influencing ODD and CD prevalence worldwide have been investigated in a systematic review and meta-regression analysis [16]. The authors have assessed all the investigations conducted between 1987 and 2008 in their study. At the end of the assessments, 39 investigations were found to meet the inclusion criteria and among them, 25 studies with available data were included in the review. The pooled prevalence of ODD was estimated to be 3.3%. The authors reported age as the only covariate that remained significantly associated with heterogeneity of results for ODD after the successive deletion of non-significant variables in the multivariate meta-regression model. Geographical area was not found to be related with estimated ODD prevalence. However, it is important to note that only 4 of the 25 studies were from non-western countries (3 from Asia, 1 Middle East) and the remaining 21 were from Europe and United States.

One of the most important gaps in the epidemiology of ADHD and ODD is the fact that age related changes of these disorders were not thoroughly investigated in longitudinal community studies [17]. It is known that ADHD persists through adolescence and adulthood and 15-80% of children diagnosed with ADHD continue to have the disorder in adulthood, depending on the diagnostic criteria used [9,18] However, these findings are mostly derived from follow-up studies relying on clinical samples of children with ADHD. Recently, Ramtekkar et al. [19] assessed a huge community sample of subjects from different ages documenting a decline of ADHD symptoms and consequently prevalence rates during the life cycle. However, this was a cross-sectional study. Similar to ADHD, longitudinal prevalence of ODD has been very rarely investigated in epidemiological sample. Nock et al. [11] suggested that ODD mean persistence is around 6 years. However, many adults diagnosed as ODD during childhood and adolescence continues to show ODD symptoms, although diagnostic criteria of ODD are not currently used in adulthood [11].

This study was conceptualized to provide the following specific data lacking in the literature: Firstly, to find out the prevalence rates of ADHD and ODD in a non-referred representative sample from a country in a region where no previous rates are available (Turkey). Turkey has also a specific importance with an interesting geographical position as a bridge between Europe and Asia. Secondly, to evaluate the differences in ADHD and ODD prevalence rates between four different waves with one-year gap in reassessments. So, the data obtained from the study has the potential of increasing our knowledge on the developmental epidemiology of ADHD and ODD.

## Method

This study is a 4-year longitudinal ADHD and ODD prevalence investigation. The first year assessment started with a screening phase in April 2008 when the ADHD and Disruptive Behavior Scales were sent to parents and teachers. A study sample was derived at the end of the screening phase and clinical evaluations (screening positive cases and matched controls) were performed in May 2008, configuring the first wave of the study. The sample was reassessed in May 2009, May 2010 and May 2011 in the second, third and fourth waves of the study.

### Sample and screening procedures

#### Sample

This study has been performed in the central district of İzmir, which is the third biggest city of Turkey. The central district of İzmir had a total population of 782.309 inhabitants, 67 government primary schools (from prep class to 8^th^ grade), a total number of students of about 155.266 and the total number of 2^nd^ graders was 12.667.

The study sample size was calculated to detect ADHD and ODD prevalence with a precision of 1%, an alpha error of 5%, and assuming both ADHD and ODD prevalence around 5%. Thus, the sample size needed for this study was estimated to be 1500 students. Sixteen schools were enrolled by a randomized sampling method among the total of 67 schools in, İzmir stratified according to the socioeconomic class categorization of the Ministry of National Education Izmir Provincial Directorate. Among these 16 schools, 1500 cases were selected again with a randomized sampling method. Parents provided written informed consents for the participation of their children and the necessary legal approvals and Universities Ethical Committee approval were obtained.

#### Screening procedures

The Turgay DSM-IV Disruptive Behavior Disorders Rating Scale (T-DSM-IV-S) was used as a screening instrument in this study. The items in the scale are identical to the list of symptoms described in the DSM-IV criteria for ADHD (inattention: 9 items, hyperactivity-impulsivity: 9 items), ODD (8 items) and CD (15 items). The T-DSM-IV-S was developed by Turgay [20] and translated and adapted in Turkish by Ercan et al. [21]. The symptoms are scored by assigning a severity estimate for each symptom on a 4-point Likert-type scale (namely, 0 = not at all; 1 = just a little; 2 = much; and 3 = very much). Ratings of “much” and “very much” for each item were considered positive, as done in other similar investigations. Scales derived from DSM-IV diagnostic criteria for ADHD, such as the AD/HD Rating Scale IV, have shown adequate criterion-related validity and good reliability in different cultures both for parent and teacher reports [22,23]. The same is true for scales derived from DSM-IV diagnostic criteria for ODD, such as SNAP-IV ODD [24,25].

T-DSM-IV-S was sent to the parents and teachers of the 1500 randomly selected cases. Cases with completed forms were admitted to the study. The cases with incomplete parent or teacher scales were excluded. Parent and teacher forms of 1455 cases were taken back and considered valid, making the response rate 97%.

For the screening of ADHD; students with at least 5 symptoms of inattention and or hyperactivity/impulsivity on both teacher and parent scales were considered to have positive screening for ADHD. This method was called as “and rule” and it is known to be a conservative approach. This cutoff point was reported to have a high sensitivity (82%) and the highest negative predictive power (96%) for the screening of ADHD in a previous study [26]. All positively screened subjects and their parents were invited to participate in the second stage of the study (N = 86).

Similarly, ODD screening relied on the “and rule” method. Students with at least 3 symptoms of ODD on both teacher and parent scales were considered to have positive screening for ODD. The aim was again to decrease the false negative ratio. In addition, some investigators suggested that the threshold of 3 symptoms might be enough for ODD diagnosis [10,27]. Like the ADHD screening positive cases, all ODD screening positive cases were invited to participate in the second stage of the study (N = 43).

Among the ADHD screening positive cases (n = 86) and ODD screening positive cases (n = 43), 59 cases were only screening positive for ADHD, 16 cases were only positive for ODD and 27 cases were positive for both ADHD and ODD. The control groups for ADHD and ODD screening positive cases were constituted by screening negative children individually matched for sex and parental education level. For ADHD, all cases both in the study group and with one exception in the control group were enrolled into the study, totalizing 171 cases (86 positive screening, 85 negative screening). One screen negative case was excluded since his parents did not want to participate in the study. In the ODD group, all the study and control group cases agreed to participate in the study, so totally 86 cases were recruited for the study (43 screening positive, 43 screening negative). Since 27 cases were positive for both ADHD and ODD, the total number of cases that were enrolled into the study was 203 (screening positive cases: 86 + 43-27 = 102; screening negative cases: 86 + 43-27-1 = 101 and total sample: 102 + 101 = 203) Since the sample of cases that matched for sex and parental education with the screen positive cases was larger; a random sample was selected among them to serve as controls.

### Diagnostic procedures

Positive and negative screening cases for ADHD and ODD diagnoses completed clinical assessments that included the Schedule for Affective Disorders and Schizophrenia for School Age Children Present and Lifetime version (K-SADS-PL) [28]. The K-SADS-PL is a highly reliable semi-structured interview for the assessment of a wide range of psychiatric disorders. Turkish reliability and validity study of K-SADS-PL was conducted by Gokler et al. [29]. Cognitive evaluation relied on the Vocabulary and Block Design subtests of the WISC-R [30] that was administered by a trained psychologist to estimate the child’s overall IQ. Parents and teachers were interviewed by a study nurse to assess the presence of impairment criterion.

In the evaluation of impairment criteria, firstly parents were interviewed about 4 areas: the child’s relationships with his/her sibling(s), friends, ability to do his/her homework and general adjustment at home. In the interviews with teachers, again 4 domains were evaluated: Whether the patient was considered problematic or not, his/her relations with friends at school, his/her general success in subjects and lastly the self-esteem level of the children. The case was considered to be impaired if he/she was rated as very problematic in at least one area or a little problematic in two or more areas. Similar approaches were previously used in other epidemiologic studies [31,32].

All the original interviews were made blind to the screening status of the cases. “A best estimate procedure” was used to determine final diagnoses [33]. “Best estimate procedure” is defined here as determining diagnostic status after reviewing all teacher and parent’s scales, semi-structured interviews conducted with parents and children (K-SADS-PL), WISC-R results, and the evaluation of impairment criterion by an independent interviewer in separate interviews conducted with teachers and parents.

In the 2^nd^, 3^rd^ and 4^th^ waves of the study, subjects from both the study and control groups of the first year were called back for reevaluation in the same school settings. All the procedures of the original study except than WISC-R were performed to the study participants by the same researchers in these waves.

In the ADHD group, 3 cases from the study group and 2 cases from the control group could not be reached in the 2nd wave (response rate of the 2nd year of the study was determined to be 97%). In the 3rd wave, same 3 cases from the study group could not be reached again but the two cases of the control group who were not reached in the 2nd wave were reached this time, making the total number of cases evaluated in the 3rd wave 168 (response rate = 98%). In the 4th wave, 5 cases from the study group and 4 cases from the control group could not be reached that decreased the response rate to 94.7%.

In the ODD group, 3 cases from the study group could not be reevaluated in the 2nd wave because their parents refused to take part in this wave; making the total number to be 83. However, all the cases including the 3 excluded cases of the 2nd wave agreed to take part in the 3rd wave and the entire original sample were assessed. Three cases from the study group and 2 cases from the control group could not be reached in the 4^th^ wave. Thus the response rates in the 2^nd^, 3^rd^ and 4^th^ waves of the study were 96.5%, 100% and 94.2% respectively.

## Data analysis

Prevalence rates of ADHD and ODD were calculated according to a standard formula as suggested by Rohde et al. [26]: Pe = cd + nns (1-npv)/n, where Pe is the estimated prevalence, cd are the cases during the diagnostic phase, nns is the number of negative screenings that were not assessed at diagnostic phase, npv is the negative predictive value of screening instrument and n is the sample size. The negative predictive value is the number of times that the screening instrument said that the subject was a non-ADHD case and the diagnostic assessment confirms the subject as a non-ADHD case. The subtraction of 1-negative predictive value gives the percentage of correct diagnosis in screening negatives. Thus, multiplying per number of screening negatives that were not assessed in diagnostic phase gives the number of potential real cases that were not assessed because they were screening negatives but have ADHD. Confidence interval for the estimated prevalence of ADHD and ODD was calculated with Fleiss Quadratic approximation method [34]. For the statistical analysis, chi square test for categorical variables and paired sample t test for numeric variables were used. P values less than 0.05 were accepted to be statistically significant.

## Results

The cases were included in analyses only if both the parent and teacher scales were returned and if they were correctly filled out. Thus out of 1500 children, 1455 (between 8 to 12 years of age) were included in the screening phase and the response rate was found to be 97% (43.1% of the subjects were females and 56.9% males).

### Prevalence rates

Using a best estimate procedure, 89 subjects were diagnosed as having ADHD (82 of these cases were from positive, and 7 of them were from negative screening group) in the wave 1. In the second wave of the study, 68 subjects from screen (+) group and 7 subjects from screen (−) group were diagnosed as ADHD (total = 75 subjects); in the third wave, 65 subjects from screen (+) group and 7 subjects from screen (−) group were diagnosed to be ADHD (total = 72 subjects) and in the fourth wave, 53 subjects from screen (+) group and 8 subjects from screen (−) group were diagnosed to be ADHD (total = 61 subjects). Prevalence rates in each wave were calculated as the number of cases detected in the diagnostic phase + number of screening negatives that were not assessed in diagnostic phase × (1 – negative predictive value)/sample size. The prevalence rates were calculated in such a complex way, because simply dividing the number of cases by the sample size would not count the cases that have the diagnosis when the screening was negative. In other words, since no screening test has 100% accuracy, there are always false negatives. The formula presented adjusts prevalence for the performance of the screening instrument. So, ADHD prevalence was calculated to be 13.38% (95% CI = 11.75-15.43) in the first wave, 12.53% (95% CI = 11.02-14.53) in the second wave, 12.22% (95% CI = 10.77-14.18) in the third wave and 12.91% (95% CI = 11.48-16.44) in the fourth wave.

For ODD, 31 cases (29 from positive screening group and 2 from negative screening group) were diagnosed to be ODD in the first wave. Since DSM-IV does not allow ODD diagnosis in the presence of conduct disorder; 8 cases with both ODD and CD diagnosis (“inclusive ODD diagnosis”) were subtracted from the total number; leaving 23 cases (22 from positive screening, 1 from negative screening group) with DSM-IV diagnosis of ODD. In the second wave, 22 of the first 23 ODD (+) cases and 61 of 63 ODD (−) cases could be assessed and among these, 9 ODD (+) cases were still found to be ODD. Five new cases from ODD (−) group in the first wave were diagnosed to be ODD in the second wave making the total number equal 14. None of the screen negative cases were diagnosed as ODD in the second wave of the study. In the third wave, all the subjects of first wave were reached and among them 13 subjects from the positive screening group were diagnosed as ODD. In the third wave of the study, 2 subjects from the negative screening group was detected to be ODD making a total number of 15 subjects to be ODD (+) (10 of these 15 subjects had been assessed as ODD (+) in the first wave and other 5 were new diagnosis). In the fourth wave of the study, 9 subjects from screen (+) group and 2 subjects from screen (−) group were diagnosed as ODD (total = 11 subjects; 6 of these 11 subjects had been assessed as ODD (+) in the first wave).

ODD prevalence was found to be 3.78% (95% CI = 3.48-6.33) in the first year, 0.96% (95% CI = 0.95-3.64) in the second year, 5.42% (95% CI = 5.04-8.27) in the third year and 5.35% (95% CI = 4.79-10.91) in the fourth year of the study. When the findings from 4 waves were evaluated concomitantly; 13.21% of the cases were found to be diagnosed as ODD in at least one wave of the study; while 0.21% of the cases were diagnosed to be ODD in all three waves. The mean ODD prevalence of three waves was found to be 3.87%.

### Diagnostic stability among waves

It was found that 74 subjects among the ADHD (+) cases of first wave (89 cases) were still found to be ADHD (+) in the second wave (3 cases were not reached). One subject who was ADHD (−) in the first wave received the ADHD diagnosis, making the total number of ADHD diagnosed cases 75 in the second wave. Thus 86% of the ADHD (+) cases of first wave were still ADHD (+) in the second wave. In the third wave, 70 subjects among the ADHD (+) cases of first wave were still found to be ADHD (+) (3 cases were not reached). Two subjects who were ADHD (−) in the first wave received ADHD diagnosis making the total number of ADHD diagnosed cases 72 in the third wave. Thus 81.4% of the ADHD (+) cases of first wave were still ADHD (+) in the third wave. In the fourth wave, 57 subjects among the ADHD (+) cases of first wave and 4 cases of ADHD (−) in the first wave were diagnosed as ADHD.

Kappa values to estimate test-retest reliability for ADHD diagnosis were found to be: a) 0.84 between the first and second waves; b) 0.78 between the first and third waves; c) 0.62 between first and fourth waves; d) 0.85 between the second and third waves; e) 0.70 between second and fourth waves and f) 0.69 between third and fourth waves (see Table 1).

**Table 1:** Distribution of ADHD and ODD diagnosis among cases according to waves

	**ADHD diagnosed cases**			**ODD diagnosed cases**		
**Screening**	**Diagnosis (n)**		**Total**	**Diagnosis (n)**		**Total**
	**(+)**	**(−)**	**(n)**	**(+)**	**(−)**	**(n)**
	***1st WAVE***					
**Screening (+)**	82	4	86	22	21	43
**Screening (−)**	7	78	85	1	42	43
**Total**	89	82	171	23	63	86
	***2nd WAVE***					
**Screening (+)**	68	15	83	14	26	40
**Screening (−)**	7	76	83	0	43	43
**Total**	75	91	166	14	69	83
	***3rd WAVE***					
**Screening (+)**	65	18	83	13	30	43
**Screening (−)**	7	78	85	2	41	43
**Total**	72	96	168	15	71	86
	***4th WAVE***					
**Screening (+)**	53	28	81	9	31	40
**Screening (−)**	8	73	81	2	39	41
**Total**	61	101	162	11	70	81

*ODD* Opposition defiant disorder, *ADHD* Attention Deficit Hyperactivity Disorder.

Around 41%, 43.5% and 27.3% of ODD cases diagnosed in the first wave were still diagnosed to be ODD in the second and third waves respectively. Kappa values to estimate the diagnostic test-retest reliability of ODD were found to be a) 0.37 between the first and second waves; b) 0.40 between the first and third waves and c) 0.22 between the first and fourth waves (see Table 1).

### Comorbid diagnosis

As expected, the main comorbid diagnoses for ADHD in the four waves were ODD: 61.8%, 45.3%, 40.8%, 39.3; CD: 18.0%, 17.3%, 12.7%, 24.6%; anxiety disorders: 32.6%, 12.0%, 5.6%, 4.9%; mood disorders: 12.4%, 2.7%, 9.9%, 11.5%; tic disorders: 6.7%, 6.7%, 6.9%, 8.2%; enuresis 19.1%, 8.0%, 4.2%, 3.3%, and encopresis 9.0%, 1.3%, 0%, 0% respectively. In addition, also as expected, the main comorbid diagnose for ODD in the four waves was ADHD: 87.0%, 92.9%, 100%, 72.7%. The comorbid diagnoses according to waves are given in Table 2 and Table 3.

**Table 2:** Distribution of comorbid diagnoses among ADHD (4) and ADHD (-) cases according to waves

	**1**^**st **^**wave**		**2**^**nd **^**wave**		**3**^**rd **^**wave**		**4**^**th **^**wave**	
	**ADHD (+)**	**ADHD (−)**	**ADHD (+)**	**ADHD (−)**	**ADHD (+)**	**ADHD (−)**	**ADHD (+)**	**ADHD (−)**
	**N = 89**	**N = 82**	**N = 75**	**N = 91**	**N = 72**	**N = 96**	**N = 61**	**N = 101**
	n-%	n-%	n-%	n-%	n-%	n-%	n-%	n-%
ODD	55–61.8%^*^	6–7.3%^*^	34–45.9%^****^	5–5.4%^****^	29–40.3%^*^	7–7.3%^*^	**24–39.3%**	8–7,9%^*^
CD	16–18.0%^*^	1–1.2%^*^	13–17.6%^****^	3–3.3%^****^	9–12.5%^*^	--^*^	**15–24,6%**	1–1%^*^
Anxiety Disorders	29–32.6%	18–22%	9–12.2%	6–6.5%	4–5.6%	6–6.3%	3–4.9%	9–8.9%
Mood Disorders	11–12.4%	5–6.1%	2–2.7%	1–1.1%	7-9.7%^***^	2.2.1%^***^	**7–11.5%**	1–1%^*^
Tic Disorders	6–6.7%	4–4.9%	2–2.7%	--	1–1.4%	--	5–8.2%	--
Enuresis	17–19.1%	11–13.4%	6–8.1%	5–5.4%	3-4.2%	3–3.1%	2–3.3%	1-1%
Encopresis	8–9.0%^**^	1–1.2%^**^	1–1.4%	1–1.1%	--	1–1%	--	--

*The comorbidity rates presented are based in the independent sub-samples assessed for ADHD. So, ODD comorbid rates does not match the number of ODD cases assessed in the ODD sub-sample.

*ODD* Opposition defiant disorder, *CD* Conduct Disorder, *ADHD* Attention Deficit Hyperactivity Disorder.

N = Number of patients with or without ADHD diagnosis, n = number of patients with comorbid diagnosis, % = Percentage of patients with comorbid diagnosis.

Bold values mark statistically significant difference.

^*^p < 0.001, ^**^p = 0.023, ^***^p = 0.034.

**Table 3:** Distribution of comorbid diagnoses among ODD (+) and ODD (−) cases according to waves*

	**1**^**st **^**wave**		**2**^**nd **^**wave**		**3**^**rd **^**wave**		**4**^**th **^**wave**	
	**ODD (+)**	**ODD (−)**	**ODD (+)**	**ODD (−)**	**ODD (+)**	**ODD (−)**	**ODD (+)**	**ODD (−)**
	**N = 23**	**N = 63**	**N = 14**	**N = 69**	**N = 15**	**N = 71**	**N = 11**	**N = 70**
	n-%	n-%	n-%	n-%	n-%	n-%	n-%	n-%
ADHD	20–87.0%^*^	20–31.7%^*^	13–92.9%^**^	32–46.4%^**^	15–100%^*^	30–42.3%^*^	**8–72.7%**	20–28.6%^*^
Anxiety Disorders	7–30.4%	26–41.3%	2–14.3%	5–7.2%	--	2–2.8%	--	4–5.7%
Mood Disorders	2–8.7%	6–9.5%	--	1–1.4%	1-6.7%	4–5.6%	--	3–4.3%
Tic Disorders	2–8.7%	4–6.3%	1–7.1%	1–1.4%	--	1–1.4%	1–9.1%	--
Enuresis	3–13.0%	12–19.0%	2–14.3%	6–8.7%	--	3–4.2%	--	2–2.9%
Encopresis	3–13.0%	1–1.6%	--	--	--	--	--	--

*The comorbidity rates presented are based in the independent sub-samples assessed for ODD. So, ADHD comorbid rates does not match the number of ADHD cases assessed in the ADHD sub-sample.

*ODD* Opposition defiant disorder, *ADHD* Attention Deficit Hyperactivity Disorder.

N = Number of patients with or without ODD diagnosis, n = Number of patients with comorbid diagnosis, % = Percentage of patients with comorbid diagnosis.

Bold values mark statistically significant difference.

^*^p < 0.001, ^**^p = 0.001.

### ADHD and ODD correlates

Male predominance was seen in both ODD and ADHD groups in all three waves of the study although statistical significance was not obtained (male/female ratio in ADHD = 3.2, 3.4, 3.5, 5.8 respectively in each wave. Male/female ratio in ODD = 10.5, 6, 14, respectively in each wave). Moreover, no significant difference was found between the ADHD (+) and (−) cases and ODD (+) and (−) cases in socio-demographic variables (parent education and neighborhood) in any wave (data available upon request). Finally, no significant difference was found between the ADHD (+) and ADHD (−) cases and ODD (+) and ODD (−) cases in estimated IQs. (Mean estimated IQ for ADHD (+) cases: 78.60 (SD: 26.70), for ADHD (−) cases: 86.06 (SD: 24.68); t = −1.864, df = 164, p = 0.064. Mean estimated IQ for ODD (+) cases: 86.14 (SD: 29.03), for ODD (−) cases: 89.52 (SD: 28.59); t = −0.477, df = 83, p = 0.635).

## Discussion

This study is a 4-year longitudinal investigation on the ADHD and ODD prevalence rates conducted in Turkey which is an interesting geographical area standing on the intersection of Europe, Asia and Middle East. It is important to note that these 3 areas have very few investigations on the prevalence of child mental disorders including ADHD and ODD. Our findings on ADHD for the 4 waves (consecutively 13.38%, 12.53%, 12.22% and 12.91%) were remarkably higher than the worldwide pooled childhood prevalence of ADHD (6.48%) [7]. On the other hand, the prevalence rates of ODD were found to be 3.77%, 0.96%, 5.41%, 5.35% respectively in the first, second, third and fourth waves and the mean ODD prevalence was found to be 3.87% which was surprisingly very close to the worldwide pooled prevalence of ODD (3.3%) [16].

The five main findings from this study were: 1) A substantially higher prevalence of ADHD in school-age children in Turkey compared to the one reported in a recent meta-analyses including studies from several different countries [7]. This difference becomes more important when we consider that diagnostic procedures followed in our study included even an independent measure of impairment; 2) The consistency of the 4-year average ODD prevalence with the literature, in spite of the fluctuations between the waves; 3) Remarkable true positivity of screening for ADHD (high positive predictive value), as well as clear stability of the diagnosis and reliable Kappa values throughout the 4 consecutive assessments with one year intervals; 4) Lower true positivity of screening for ODD and less stable ODD diagnosis with smaller Kappa values throughout the 4-years interval compared to ADHD; 5) much larger prevalence of ODD among boys than girls.

In Turkey, ADHD prevalence was not previously studied in extensive investigations, but two studies with diagnostic procedures including only parent and teacher scales reported the prevalence of ADHD in school age children between 6–12 and 6–15 years as 8.1% and 8.4 respectively [35,36]. It was interesting to find a higher ADHD prevalence in spite of the use of stricter methodology in our study. Moreover, ODD and ADHD prevalence rates were determined with same methodology but only ADHD prevalence rates were found to be high throughout the 4 waves. The ODD prevalence was found to be much less than the previous studies conducted in Turkey that was based on scale assessments [35,36]. These results may raise the question “whether ADHD is more prevalent in Turkish children than some other parts of the world?” Considering the migratory origin of Turkish people, which was officially registered with “sella turcica” in anatomy, and the relation between migration and DRD4 gene (one of the most important candidate genes in ADHD etiology) this hypothesis worth to be investigated [37].

One of the most important aspects of ADHD epidemiology is the course of the disorder across the life span. The ADHD prevalence tends to decrease with age and 60% of the cases diagnosed as ADHD during childhood continue to be diagnosed with ADHD in adulthood [38]. But most of the data supporting this idea is derived from follow-up studies of clinical samples. There are scarce data from longitudinal non-referred samples [17]. In the study of Cohen et al. [39] which was an 8 year follow-up study, ADHD prevalence was found to be 12.8%, 9% and 6% in the same cohort in the following age ranges: 10–13, 14–16 and 17–20 year. In another study conducted in Spain among children at 8, 11 and 15 years old, ADHD prevalence was found to be 14.4%, 5.3% and 3%, respectively [40], and in another study 6.8% in the same country [41]. A study from Canada, reported ADHD prevalence as 5.5%, 4% and 2.5% among 6–8, 9–11 and 12–14 years-old subjects, respectively [42]. When the results of these studies are evaluated, a 0.5 to 1% decrease in ADHD prevalence rates in average is found for each 1-year increase in age. In our study, we detected a decrease of 0.47% in the prevalence rates of ADHD between the first and fourth waves which is completely consistent with previous studies. The future follow up of the subjects of this study will provide more information about the lifelong prevalence of ADHD.

One interesting finding was the fluctuation of ODD prevalence rates among the three waves. It has been known that variations like presence or absence of diagnosis are frequent in longitudinal studies [43]. Lavigne et al. [44] evaluated 510 children aged 2–5 years old with ODD for a period of 48–72 months at 5 separate waves and they stated that part of the fluctuations among the waves involves a move from exceeding a diagnostic threshold to a subthreshold status and vice versa. In our study these fluctuations may be explained similarly.

While the mean ODD prevalence found in this study is very consistent with the literature; distribution of the cases according to sex is quite different than previous literature findings. Female/male ratio of ODD was around 1/10 in all three waves of our study. This is a quite large difference, although statistical significance was not obtained. ODD prevalence was reported to be higher in boys than in girls in most of the previous studies, especially in preadolescent period. Among the previous literature, there is only one study with similar findings to our results in terms of male/female ODD ratio; but nevermore only in a subgroup of the study (Female/Male = 1/4) [45]. In that study, disruptive behavior disorders prevalence rates were compared among the Puerto Ricans living in Puerto Rico and New York and male–female difference was found to be quite large only among the cases between ages 5–9 living in New York; while in other residency places and age groups, large differences were not found in spite of the dominancy of males [45]. Although cultural variations may be suggested as an explanation for this result from our study, the findings from another study conducted previously in Turkey reporting a very close distribution of ODD cases among boys and girls (55.5% in boys vs. 44.5% in girls) does not support this view [36]. It is important to note that there is an active debate on the applicability of DSM-IV ODD criteria to girls [30,46]. Some authors have suggested that aggression might be expressed among girls in a different way than boys; covert aggression may be a more frequently used style among girls instead of overt aggression [47]. In terms of ADHD diagnosis, the dominance of boys compared to girls was an expected finding. The male/female ADHD ratio was 3.2 times higher in male than females in the first wave and 3.4 times higher in the second wave and 3.5 times higher in the third waves.

Parent education and neighborhood were the only investigated socioeconomic variables in the study and no statistically significant difference was found between controls and both ADHD and ODD groups. In an epidemiological survey from Germany, ADHD was reported to be more frequent among subjects with low socioeconomic status whereas there were no statistically significant differences with regard to geographical characteristics (e.g. urban vs. rural) [48]. It has been reported that ODD and CD occurs most frequent in lower socioeconomic groups [13,46]. But it has also been stated that prevalence rates of disruptive behavior disorder in the disadvantaged neighborhoods compared with advantaged inner-city neighborhoods have not been sufficiently documented, and the current evidence on possible differences in the prevalence of ODD and CD in rural and urban environments is mixed [2,13].

### Limitations

Our findings should be understood in the context of some limitations. First, this is a regional study of ADHD and ODD prevalence and the results may not generalize to other areas of Turkey. Second, we only included public schools. Therefore, cases found in the top socio-economic group may not be sufficiently represented. Thirdly, socioeconomic status was not extensively evaluated in this study. Finally, we did not assess all possible comorbidities.

## Conclusion

Our findings describe the ADHD prevalence rates and stability for the first time in a region where no previous study was conducted implementing a careful and extensive diagnostic procedure. Surprisingly, even after using strict criteria with impairment assessments, we found a high ADHD prevalence rate for children at 8 to 10 years of age. In addition, ADHD diagnosis was quite stable in reassessments one, two and three years after. Our results argues against the hypothesis that ADHD is a cultural construct that is uniquely associated with the United States or any particular culture. More studies from regions with no previous epidemiological information on ADHD will improve our knowledge on the real impact of cultural diversity in the disorder.

In addition, to the best of our knowledge, this study was the first longitudinal prevalence study which evaluated ODD in particular by assessing it separately from CD. This study was designed with the aim to investigate ODD prevalence in a non-referred school sample, with a well defined and sufficient sample size, by three stage evaluation and by considering impairment criteria and employing best estimate procedure for diagnosis. An ODD prevalence which was very consistent with the worldwide pooled prevalence was found in the study but diagnostic stability was found to be much lower compared to ADHD diagnosis. The results obtained from this longitudinal study should be confirmed with future studies using state of the art methodologies in different regions of Turkey.

### Clinical significance

The findings of the study described the ADHD prevalence rates and stability for the first time in Turkey which is a region where no previous study was conducted implementing a careful and extensive diagnostic procedure. In addition, to the best of our knowledge, this study was the first longitudinal prevalence study which evaluated ODD in particular by assessing it separately from CD. An ODD prevalence which was very consistent with the worldwide pooled prevalence was found in the study but diagnostic stability was found to be much lower compared to ADHD diagnosis.

## Competing interests

No financial or material support was taken for the study. Dr. Ercan is on advisory boards for Eli Lilly and Janssen. Dr. Rohde was on the speakers’ bureau and/or acted as consultant for Eli-Lilly, Janssen-Cilag Turkey, Novartis and Shire in the last three years (less than U$ 10,000 per year and reflecting less than 5% of his gross income per year). He also received travel awards (air tickets + hotel) for taking part of two child psychiatric meetings from Novartis and Janssen-Cilag. The ADHD and Juvenile Bipolar Disorder Outpatient Programs chaired by him received unrestricted educational and research support from the following pharmaceutical companies in the last three years: Abbott, Bristol-Myers Squibb, Eli-Lilly, Janssen-Cilag, Novartis, and Shire. The other authors declare that they have no competing interests.

## Authors’ contributions

All authors but LAR contributed equally to the design and conduct of the study, interpretation of the results, and writing of the manuscript. LAR was responsible for the methodology and statistical analysis of the data. All authors read and approved the final manuscript.
